# Natural transformation occurs independently of the essential actin-like MreB cytoskeleton in *Legionella pneumophila*

**DOI:** 10.1038/srep16033

**Published:** 2015-11-03

**Authors:** Pierre-Alexandre Juan, Laetitia Attaiech, Xavier Charpentier

**Affiliations:** 1 CNRS UMR5240 MAP, Villeurbanne, France; 2 Université Claude Bernard Lyon 1, Villeurbanne, France

## Abstract

Natural transformation is the process by which bacteria can actively take up and integrate exogenous DNA thereby providing a source of genetic diversity. Under specific growth conditions the coordinated expression of several genes – a situation referred to as “competence” – allows bacteria to assemble a highly processive and dedicated system that can import high molecular weight DNA. Within the cell these large imported DNA molecules are protected from degradation and brought to the chromosome for recombination. Here, we report elevated expression of *mreB* during competence in the Gram-negative pathogen *Legionella pneumophila*. Interestingly a similar observation had previously been reported in the distantly-related Gram-positive organism *Bacillus subtilis*. MreB is often viewed as the bacterial actin homolog contributing to bacterial morphogenesis by coordinating peptidoglycan-synthesising complexes. In addition MreB is increasingly found to be involved in a growing number of processes including chromosome segregation and motor-driven motility. Using genetic and pharmacological approaches, we examined the possible role of MreB during natural transformation in *L. pneumophila*. Our data show that natural transformation does not require MreB dynamics and exclude a direct role of MreB filaments in the transport of foreign DNA and its recombination in the chromosome.

Natural transformation is a major mechanism of horizontal gene transfer (HGT) and plays a leading role in bacterial genetic diversification^1^. In contrast to other HGT mechanisms it is inherent to the species, i.e. directly encoded in the bacterial genome. Naturally transformable bacteria can enter a genetically programmed and often transient physiological state called competence in which they can take up exogenous DNA and integrate it in their chromosome^2^,^3^. The state of competence mostly corresponds to the concerted expression of a multi-component DNA uptake system. Recent advances in the visualisation of the uptake system suggest a unified model between Gram-positive and Gram-negative bacteria^4^,^5^. The system involves the assembly of a type IV pilus exposed at the cell surface and that crosses the outer membrane of Gram-negative bacteria through the PilQ secretin. In the periplasmic space, the ComEA protein binds double-stranded DNA^6^ which is then converted into single-stranded DNA (ssDNA) and transported across the inner membrane through a putative transmembrane channel formed by ComEC^7^. The cytoplasmic ssDNA is bound by the single-stranded DNA Binding proteins SsbB and DprA that protect it from nucleases and brought to the chromosome for integration^8^,^9^.

Integration of the internalized ssDNA into the chromosome requires a core and conserved process, homologous recombination. While RecA is primarily involved in the maintenance of chromosome integrity it is also needed to recombine the internalised DNA with the chromosome through the interaction with the transformation-specific protein DprA^8^. In all transformable species *recA* is essential for transformation even when not part of the competence regulon, as for instance in *Haemophilus influenzae*^10^,^11^ and *Vibrio cholerae*^4^,^12^. Moreover, the importance of its increased expression during competence development has been demonstrated in the transformable species *Streptococcus pneumoniae* and *Bacillus subtilis*^13^,^14^,^15^. Although not an absolute rule, co-regulation can indicate a functional relationship and competence co-regulated genes may pinpoint fundamental processes on which cells rely for natural transformation.

DNA uptake depends on the proton motive force^16^ and the entropic forces associated with ComEA binding^17^ but the mechanism that guides the large imported DNA molecules, up to several tens of kilobases, to the nucleoid for recombination remains elusive^18^. While investigating regulation of competence in the Gram-negative human pathogen *Legionella pneumophila* we stumbled upon the observation that *mreB* is strongly induced during competence. Our data are consistent with the induction of *mreB* and its paralog *mbl* observed during competence in the distantly-related Gram-positive *Bacillus subtilis*^19^. MreB is a prominent member of the family of bacterial actins^20^,^21^. MreB assembles into filaments located below the inner membrane and determines the shape of rod-shaped bacteria by coordinating the movement of peptidoglycan-synthesising enzymatic complexes^22^,^23^. Although controversial^24^,^25^, several studies have involved MreB dynamics in chromosome segregation^26^,^27^,^28^,^29^,^30^. It is also well established that filaments of bacterial actin molecules can actively move extra-chromosomal DNA molecules within the cell to allow their effective partitioning in the daughter cells^21^,^31^. The bacterial DNA uptake system is a composite system involving a surface-exposed type IV pilus and a membrane-associated complex that allows the import of DNA as a single-strand molecule^3^,^32^. In *Pseudomonas aeruginosa* inactivation of MreB leads to mislocalisation of the type IV pilus retraction ATPase PilT^33^ suggesting that MreB might also be important for the functioning of the transformation pilus. Taken together the data strongly suggested a direct role of MreB filaments at various stage of the transport of foreign DNA during the process of natural genetic transformation. This hypothesis has recently been tested in the Gram-positive organism *B. subtilis*^34^. The data confirmed that *mreB* is induced during competence in *B. subtilis* and under control of the competence regulator ComK. MreB was found to interact with the ComGA ATPase of the DNA uptake system and this sequestration by ComGA was proposed to prevent cell elongation at the exit of competence^34^. In contrast to ComGA which is required for natural transformation^35^, MreB was found to play no role in natural transformation in *B. subtilis* as an *mreB* deletion mutant showed no defect in transformation efficiency. However, in this species, a potential involvement of the actin-like cytoskeleton in natural transformation may be obscured by the presence of two MreB paralogs (Mbl and MreBH) that can partly maintain cell growth and morphogenesis in absence of MreB^36^.

In contrast to *B. subtilis*, examination of the *L. pneumophila* genome revealed a single *mreB* gene. We found the *L. pneumophila mreB* to be expressed during competence as part of an operon with *comEA*, a gene encoding a core component of the DNA uptake system. We investigated the mechanism of induction of *mreB* during competence in *L. pneumophila* and examined a possible role of MreB in natural transformation using genetic and pharmacological approaches. Our data suggest that the co-regulation of MreB and the DNA uptake system is coincidental and that natural transformation occurs without assistance of the MreB cytoskeleton.

## Results

### *mreB* is induced during competence as a bicistronic mRNA with *comEA*

**Figure 1:**
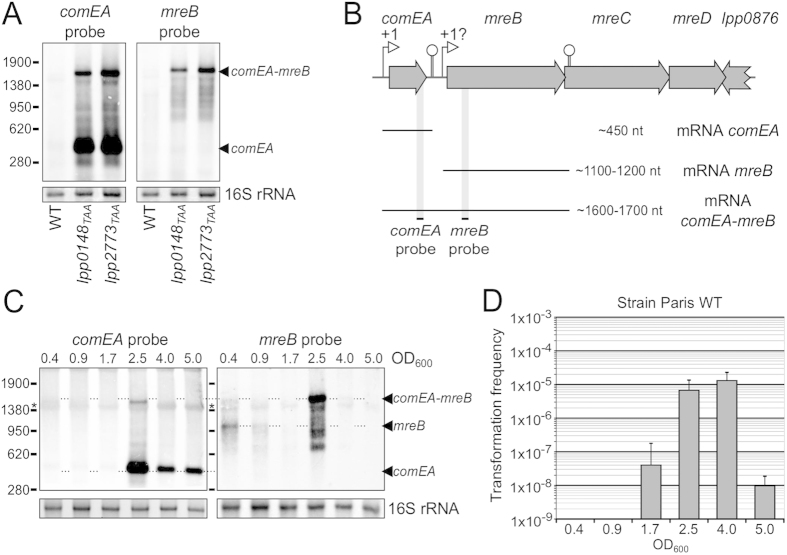
*mreB* is induced and expressed as a bicistronic mRNA during competence. (**A**) Northern-blot analyses of *comEA* and *mreB* expression in the Paris wild-type strain and in the constitutively competent mutants *lpp0148*_*TAA*_ and *lpp2773*_*TAA*_. (**B**) Schematic representation of the *comEA-mreBCD* locus in the Paris wild-type strain. Experimentally determined and annotated promoters and terminators are indicated by an open arrow and a stem-loop structure, respectively. The interrogation mark above the promoter of *mreB* indicates that the transcription start site is not precisely known. The different mRNAs produced in this region are represented by thin lines and their sizes are indicated. The black dashes and shadows show the localisation of the probes used in Northern-blot experiments. (**C**) Northern-blot analyses of *comEA* and *mreB* expression in the Paris wild-type strain during growth at 30 °C. The optical density at 600 nm (OD_600_) of the collected samples is indicated above the corresponding lanes. Ethidium-bromide staining of 16S ribosomal RNA was used to ensure that equal amounts of total RNA were loaded in each lane. The length in nucleotides and the position of the RNA ladder bands are shown on the left. The star (*) indicates non-specific binding of the Northern-blot probes on the abundant 16S rRNA transcript. (**D**) Determination of the transformation frequency in the Paris wild-type strain during growth at 30 °C. At different OD_600_ during growth, bacteria were exposed to transforming DNA for 30 minutes and then plated on selective and non-selective media. The transformation frequency is the ratio of the number of CFUs counted on selective medium divided by the number of CFUs counted on non-selective medium. At OD_600_ lower than 1.7, transformation frequency were below the detection limit (<1.10^−09^). Transformation experiments in the absence of DNA never gave rise to transformants or spontaneous resistant mutants.

Competence for natural transformation in *L. pneumophila* has previously been reported during growth under microaerophilic conditions^37^. Under standard growth conditions competence is otherwise repressed. In the *L. pneumophila* strains AA100 and JR32, mutations in the *proQ* and *comR* genes fully de-repressed competence and resulted in high levels of transformability^38^,^39^. To gain further insight into how these genes repress competence we constructed markerless mutants of *proQ* and *comR* (respectively *lpp0148* and *lpp2773*) in the Paris strain by inserting a premature ochre codon (TAA) at position 11 (see Materials and Methods). As anticipated both *lpp0148*_*TAA*_ and *lpp2773*_*TAA*_ mutants were highly transformable with a typical transformation frequency of 1.10^−4^ including under conditions where the Paris wild-type strain is not transformable (transformation frequency < 1.10^−9^) (data not shown). It was previously found that *comEA* is the most differentially expressed gene in the competent state^39^. ComEA is a small periplasmic protein involved in the DNA uptake system and required for natural transformation of *L. pneumophila*^39^. While analysing levels of the *comEA* transcript (450 nt) by Northern-blot we noticed an additional band corresponding to a larger transcript of about 1700 nucleotides (Fig. 1A). A similar band was actually visible in previously performed Northern-blot analysis of *comEA* in the kanamycin-resistant JR32 *proQ* and JR32 *comR* mutants (data not shown). An analysis of the *comEA* locus showed a small intergenic region (less than 200 bp) between the coding sequences of *comEA* and *mreB* (Fig. 1B). The *mre*B gene is predicted to form an operon with *mreC* and *mreD*, but a readthrough bypassing the terminator of *comEA* was possible. To investigate this possibility, we designed a Northern-blot probe localized in the *mreB* gene. This probe revealed a transcript of identical size and expression pattern as the large transcript revealed with the *comEA* probe (Fig. 1A). The size of the transcript is consistent with a *comEA-mreB* bicistronic mRNA starting at the mapped *comEA* promoter and ending at a transcription terminator annotated at the end of *mreB* (Fig. 1B)^40^. In contrast to the *lpp0148*_*TAA*_ mutant, the Paris wild-type strain is not naturally transformable when grown at 37 °C. However, as seen by *comEA* expression (Fig. 1C), it naturally and transiently develops competence and becomes transformable (Fig. 1D) during late exponential growth when cultured at 30 °C^41^. Under these conditions *comEA* is strongly induced when the culture reaches an optical density (OD 600 nm) of ~2.5 and a similar *comEA-mreB* mRNA is then also detectable, thereby confirming the transcriptional readthrough between these two genes (Fig. 1C). Northern-blot for the *mreB* gene shows that *mreB* is expressed as a monocistronic mRNA of approximately 1100 nt in early growth phase (OD_600_ = 0.4) and is then strongly induced to form a bicistronic mRNA when *comEA* is expressed (OD_600_ = 2.5) (Fig. 1C). We conclude that the *comEA* and *mreB* genes form a bicistronic operon induced during competence development. Operon structures are often indicative of a functional relationship. Our data suggested that MreB could have a function connected to ComEA and thus might play a role in natural transformation.

### MreB is essential in *Legionella pneumophila*

**Figure 2:**
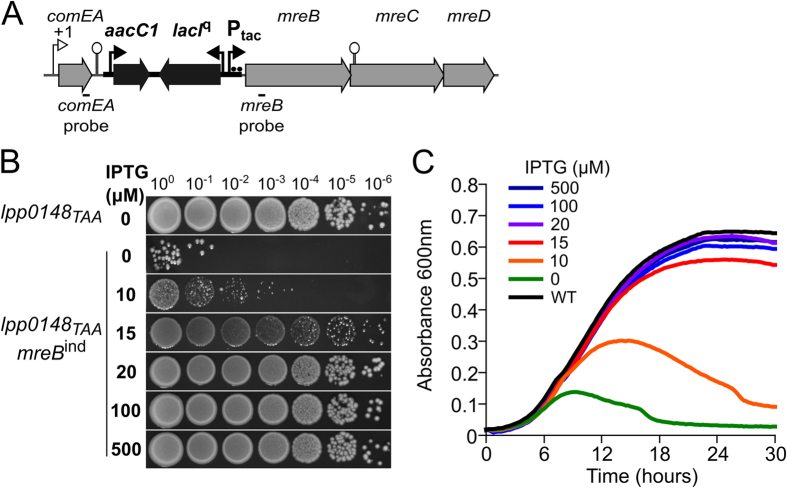
*mreB* is essential in *L. pneumophila*. (**A**) Schematic representation of the inducible allele of *mreB*. A synthetic DNA sequence carrying the gentamicin resistance gene *aacC1*, the *lacIq* gene (large black arrows) and a synthetic promoter with two LacO sites (black arrow and black dots) was inserted between the *comEA* gene and the *mreBCD* locus (large grey arrows). (**B**) Plating of ten-fold serial dilutions of the *lpp0148*_*TAA*_ *mreB*^*ind*^ strain on CYE plates containing different amount of IPTG. (**C**) Growth curve of the *lpp0148*_*TAA*_ *mreB*^*ind*^ strain in AYE in the presence of various concentration of IPTG. The culture was initiated with the *lpp0148*_*TAA*_ *mreB*^*ind*^ strain previously grown on a CYE plate with 20 μM of IPTG and resuspended in warm medium (starting OD_600_ = 0.1). Absorbance of the culture was monitored at 600 nm every 15 min with intermittent shaking in a 96-well plate reader.

In order to test a possible role of MreB in natural transformation we sought to construct a deletion mutant by replacing the *mreB* gene with a kanamycin resistance gene in the constitutively transformable *lpp0148*_*TAA*_ mutant. Repeated attempts failed to produce kanamycin-resistant transformants. As *mreB* had been found to be essential in different rod-shaped bacteria such as *B. subtilis*, *Escherichia coli* and *Caulobacter crescentus*, we thought that it could also be the case in *L. pneumophila*^42^,^43^,^44^. We thus constructed a conditional mutant of *mreB* by replacing its promoter by a synthetic IPTG-inducible promoter (IPTG = Isopropyl β-D-1-thiogalactopyranoside) at the natural chromosomal locus of the constitutively transformable *lpp0148*_*TAA*_ strain (Fig. 2A). The resulting inducible *mreB* allele will be referred to as *mreB*^*ind*^. In the presence of IPTG the *lpp0148*_*TAA*_ *mreB*^*ind*^strain was able to form colonies on CYE plates. Consistent with the low levels of *mreB* transcription in the Paris wild-type strain, low concentrations of IPTG down to 20 μM allowed normal growth (Fig. 2B). At 15 μM of IPTG, the *lpp0148*_*TAA*_ *mreB*^*ind*^ strain could not form normal size colonies and lower concentration could not support normal growth. In the absence of IPTG, revertants that escaped LacI^q^ repression were obtained at a frequency of ~1.10^−6^ (Fig. 2B). Similar results were obtained in liquid culture. The *lpp0148*_*TAA*_ *mreB*^*ind*^ strain was grown on an IPTG-containing plate and inoculated in AYE broth with various concentration of IPTG (Fig. 2C). In the absence of IPTG, the culture started to grow presumably because the cells could rely on the pre-synthesised pool of MreB. As this pool decreased in dividing cells the culture rapidly stopped growing after about two generations (the *L. pneumophila* doubling time is two hours) and then collapsed because of cell lysis. Exactly as on solid media, concentrations of IPTG above 20 μM could support normal growth (Fig. 2C). Thus, although expressed at low levels, *mreB* is required for growth and we conclude that *mreB* is an essential gene in *L. pneumophila*.

### Upregulation of *mreB* is not required for natural transformation

**Figure 3:**
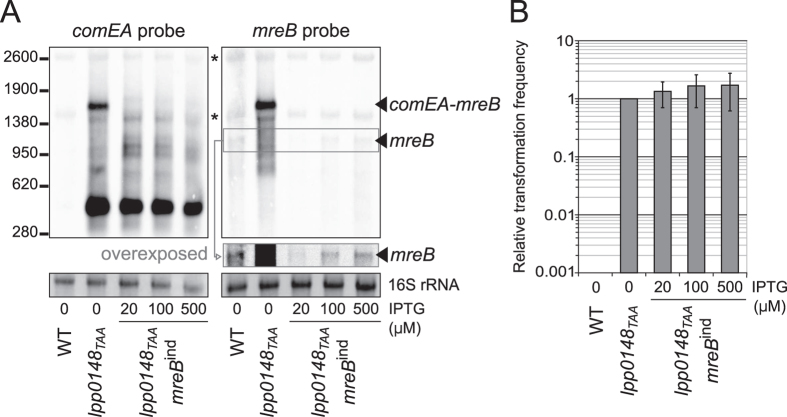
Up-regulation of *mreB* during competence is not required for natural transformation. (**A**) Northern-blot analyses of the Paris wild-type, *lpp0148*_*TAA*_ and *lpp0148*_*TAA*_ *mreB*^*ind*^strains. The strains were grown in AYE with various concentration of IPTG until the culture reached an OD_600_ of ~1. RNA were extracted, separated on a denaturing agarose gel and probed with *comEA* and *mreB* probes. Ethidium-bromide staining of 16S ribosomal RNA was used to ensure that equal amounts of total RNA were loaded in each lane. The length in nucleotides and the position of the RNA ladder bands are shown on the left. (**B**) Effect of the expression level of MreB on natural transformation. The natural transformability of the *lpp0148*_*TAA*_ *mreB*^*ind*^strain in the presence of varying concentrations of IPTG was determined under the same conditions as for panel (**A**) Bacteria were exposed to transforming DNA for 20 minutes and then plated on selective and non-selective media. Relative transformation frequency is the ratio of the number of CFUs counted on selective medium divided by the number of CFUs counted on non-selective medium. For each experiment data are expressed relative to the *lpp0148*_*TAA*_ strain. At this OD_600_ and temperature, the transformation frequency of the Paris wild-type strain is always below the detection limit (<1.10^−09^). Transformation experiments in the absence of DNA never gave rise to transformants or spontaneous resistant mutants. Depending on experiments transformation frequencies ranged from 4.10^−06^ to 1.10^−04^. Error bars represent standard deviation from the mean of at least three independent experiments.

Construction of a conditional *mreB* mutant proved that MreB is essential but also showed that low levels of MreB are sufficient for normal growth. The synchronous induction of *mreB* and *comEA* suggests that higher levels of MreB may be needed to support transformation. To test this possibility we first verified that expression of the genetically engineered inducible *mreB*^*ind*^ allele is disconnected from that of *comEA*. The *lpp0148*_*TAA*_ *mreB*^*ind*^ strain grown on CYE plate containing 20 μM IPTG was inoculated in liquid AYE medium supplemented with different concentration of IPTG and allowed to grow for two generations after which total RNAs were extracted. As expected, and because of the insertion between *comEA* and *mreB* (Fig. 2A), Northern-blot analysis of *comEA* expression shows that in the *lpp0148*_*TAA*_ *mreB*^*ind*^strain the transcript corresponding to the *comEA-mreB* mRNA is now absent (Fig. 3A). Analysis of the same samples for *mreB* expression show that, even at the highest concentration of IPTG (500 μM), *mreB* is far less expressed in the *lpp0148*_*TAA*_ *mreB*^*ind*^strain than in the *lpp0148*_*TAA*_ strain (24 ± 4 times, as determined by densitometry) (Fig. 3A). Importantly these differences in *mreB* expression levels have no dramatic consequences on the cells that show an elongated rod-shaped morphology (Fig. S1). At 500 μM of IPTG, *mreB* expression in the *lpp0148*_*TAA*_ *mreB*^*ind*^strain is similar to that in the Paris wild-type strain (1.08 ± 0.07). This condition effectively represents the situation where *mreB* is expressed at the basal level of the Paris wild-type strain independently of competence development. In this condition, the transformability of the *lpp0148*_*TAA*_ *mreB*^*ind*^ strain is equivalent to that of the *lpp0148*_*TAA*_ strain (Fig. 3B) showing that the elevated expression of *mreB* during competence is not required for DNA uptake and subsequent transformation. Moreover, it is interesting to note that, at the lowest concentration of IPTG that supports growth (20 μM) the *mreB* transcript is below the detection level which is about 40-times below the expression level in the Paris wild-type strain. Yet this did not result in a significant difference in transformability. Collectively the data suggest that the induction of *mreB* during competence is coincidental and has no functional significance with respect to natural transformation.

### Morphologically altered MreB-depleted cells remain transformable

**Figure 4:**
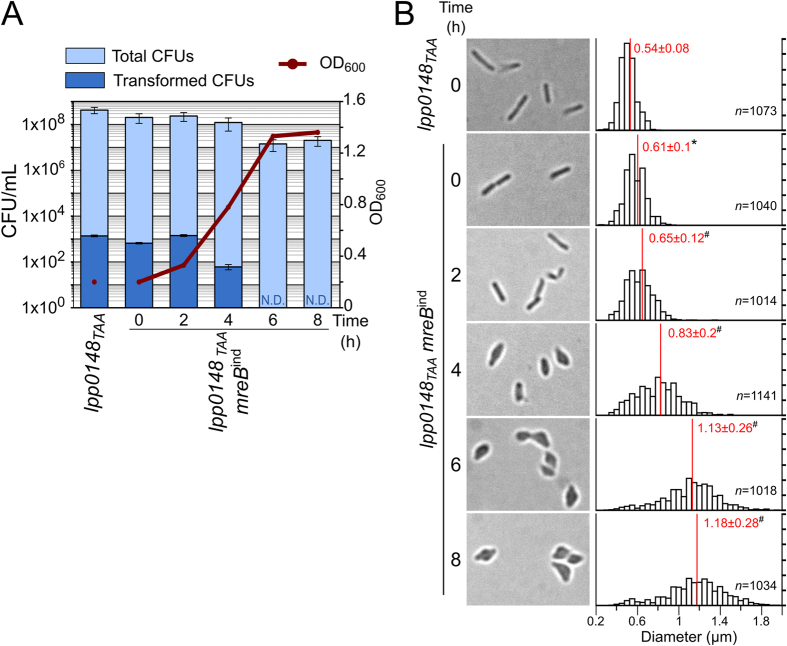
Morphologically altered MreB-depleted cells remain transformable. (**A**) Growth of the *lpp0148*_*TAA*_ *mreB*^*ind*^strain in the absence of IPTG. The culture was initiated with the *lpp0148*_*TAA*_ *mreB*^*ind*^ strain previously grown on a CYE plate with 20 μM of IPTG and resuspended in warm medium (37 °C, starting OD_600_ = 0.2). Optical density of the culture was monitored at 600 nm. Aliquots of the culture were simultaneously tested for transformability and plated on selective CYE + IPTG + Kan plates to determine colony-forming units of transformants (CFU) and on non-selective CYE + IPTG plates for determination of total CFU counts. Data are representative of three independent experiments. Error bars represent standard error of experimental determination of CFU counts. N.D., not detectable. (**B**) Bright light microscopy of bacterial cells of the cultures tested for transformability and corresponding distribution of the bacterial cells diameter measured on at least 1000 cells. The mean (red line) and standard deviation of the sample are indicated for each distribution. Statistical analysis was performed on the different data sets (see Material and Methods). A Shapiro-Wilk test confirmed the Normal distribution of the measured parameter for each condition (p < 0.01). A Welch’s t-test determined that the mean cell diameter of the *lpp01048*_*TAA*_ *mreB*^*ind*^ T0 sample is significantly different (p < 0.01) from that of the *lpp01048*_*TAA*_ strain (black asterisk). Similarly the mean cell diameter of each *lpp01048*_*TAA*_ *mreB*^*ind*^ sample (T_N_, with N the number of hours) is significantly different (p < 0.01) from that of the *lpp01048*_*TAA*_ *mreB*^*ind*^ T_N-2_ sample (black hash mark).

The above experiments could not rule out the possibility that the low levels of MreB required for growth could be also needed to import and/or integrate exogenous DNA. As described above, the *lpp0148*_*TAA*_ *mreB*^*ind*^strain can be reproducibly grown in the absence of IPTG for two generations before growth begins to slow and then stops (Fig. 2C and Fig. 4A). This provided a narrow window during which we could observe the consequences of a gradual loss of MreB in dividing cells including on natural transformability. The *lpp0148*_*TAA*_ *mreB*^*ind*^strain was grown for 24 h on CYE plate with the least amount of IPTG that could support growth (20 μM). The cells were then transferred to liquid IPTG-free medium at an OD_600_ of 0.2 and growth was monitored along with cell morphology. In agreement with the two hours generation time of *L. pneumophila*, at 2 hours post-inoculation the OD_600_ had doubled and the bacteria retained their elongated rod shape but the mean cell diameter (0.65 μm) was significantly increased compared to their initial mean cell diameter (0.61 μm) (Fig. 4B). Another two hours later (T = 4 h) the bacteria already showed strong alterations in their rod-shaped morphology and appeared enlarged at mid-cell (mean diameter of 0.83 μm) which may have contributed to the increase in OD_600_. Indeed, despite increased OD_600_ the viable count determined on CYE plates containing IPTG remained steady. At the next generation time, the bacteria displayed a dramatically altered morphology with an ellipsoidal to ovoid shape (mean diameter = 1.13 μm) and their viability was strongly affected (Fig. 4A,B). At T = 2 h, the cells could still divide but the MreB levels were already too low to properly coordinate cell wall synthesis resulting in visible defect on the next generation time (at T = 4 h) but transformation seemed unaffected (Fig. 4A). Beyond T = 6 h, cells could no longer divide and no transformants could be obtained. It is of note that the decreased viability at this time point prevented the detection of transformants occurring at a frequency below 1.10^−7^. At T = 4 h the defects in cell morphology indicate that peptidoglycan assembly had been going on in the absence of functional shape-determining MreB cytoskeleton. Although moribund and deformed these cells remained transformable. At this time point, transformation frequency (5.07 × 10^−7^) is reduced about 6-fold when compared to that of the *lpp0148*_*TAA*_ *mreB*^*ind*^ cells at T = 0 (3.29 × 10^−6^) or to the *lpp0148*_*TAA*_ cells having competence-induced levels of MreB (3.26 × 10^−6^). This decreased transformability appears somehow limited given the poor state of the cells at this stage and a requirement of MreB for the functioning of the DNA uptake system can be excluded. Yet since transformation frequency is reduced a minor role cannot be ruled out. Alternatively this limited decrease in transformability of MreB-depleted cells may represent an indirect effect of the major disturbance of the cell wall that non-specifically alter the assembly and/or localisation of membrane-associated systems^33^,^45^,^46^.

### Natural transformation is resistant to the MreB inhibitor A22

**Figure 5:**
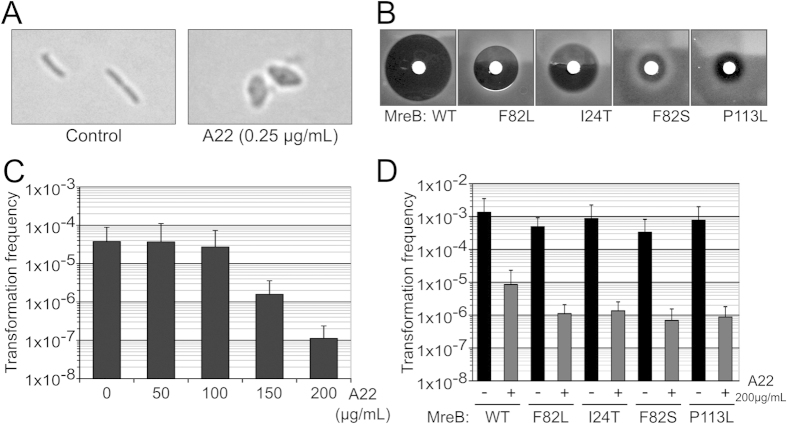
Natural transformation is resistant to the MreB inhibitor A22. (**A**) Bright light microscopy of *L. pneumophila* chronically exposed to low levels of A22 (0.25 μg/mL). (**B**) Sensitivity to A22 of A22-resistant mutants of MreB determined by disc diffusion assay. (**C**) Determination of the natural transformability of the *L. pneumophila lpp0148*_*TAA*_ strain in the presence of increasing concentrations of A22. Transformation frequency is the ratio of the number of CFUs counted on selective medium divided by the number of CFUs counted on non-selective medium. Error bars represent standard deviation from the mean of three independent experiments. (**D**) Effect of high concentration of A22 on the transformability of A22-sensitive and A22-resistant strains. Error bars represent standard deviation from the mean of three independent experiments.

In order to ascertain a possible role of MreB in natural transformation without altering viability or inducing major disturbance of the cell morphology we sought to use MreB inhibitors. A22 is a widely used small molecule inhibitor^47^ that interacts with the MreB nucleotide binding pocket^48^. In several species (*Caulobacter crescentus*, *Escherichia coli*, *Pseudomonas aeruginosa*, *Myxococcus xanthus*), A22 was shown to cause a rapid and reversible disturbance of the MreB cytoskeleton thereby arresting MreB-dependent processes within one to two minutes without immediately affecting cell morphology^28^,^33^,^49^,^50^. The effect of MreB inhibition on cell morphology only becomes apparent when the cells are allowed to grow while MreB cytoskeleton is disrupted, as they then display a round-shaped morphology. Consistent with the essential nature of MreB in *L. pneumophila* A22 showed antibiotic activity against this organism and cells growing in growth-limiting sub-inhibitory concentration of A22 (0.25 μg/mL) exhibit a morphology identical to MreB-depleted cells (Fig. 5A). We isolated twelve mutants that were resistant to various levels of A22. All mutants were found to have acquired non-synonymous mutations in the *mreB* gene associated with various A22-resistance levels (Fig. S2). Using the *lpp0148*_*TAA*_ *mreB*^*ind*^strain we could transfer four of the most resistant alleles to a genetic background previously unexposed to A22 (see Materials and Methods) and verified that these mutations were indeed sufficient to confer resistance to A22 (Fig. 5B). These data confirmed that MreB is the primary target of A22 in *L. pneumophila* and that it alters the MreB cytoskeleton similarly to as in other organisms. We therefore tested transformability against a wide range of A22 concentrations. At concentrations up to 100 μg/mL, which is well above the MIC, we did not observe any effect on natural transformation (Fig. 5C). Treatment of the bacteria with A22 for 5 minutes prior to transformation did not reduce transformability either (data not shown). However at higher concentrations, A22 exerted a significant effect on natural transformability, reducing it by up to two orders or magnitude (Fig. 5C). Yet transformability of A22-resistant MreB mutants was equally affected suggesting that inhibition of transformation at high concentrations results from an off-target effect (Fig. 5D). We conclude that natural transformation is resistant to A22-mediated inhibition of MreB and thus that MreB does not play any direct role in natural transformation.

## Discussion

In eukaryotes actin filament structures that control the shape of the cell are also used in other non-essential cellular processes (*i.e.* phagocytosis) and even subverted by pathogens. A similar situation exists with MreB, although its primary function is to coordinate cell wall synthesis it can also serve other active processes such as gliding motility^50^,^51^, type IV pilus-dependent twitching motility^33^ and infection of bacterial virus^52^,^53^. Like twitching motility, natural transformation is associated with type IV pili, and import of DNA macromolecule represented a likely MreB-dependent process. Another compelling evidence for a possible role of MreB in natural transformation came from the observation that *mreB* is up-regulated during competence in the Gram-positive *B. subtilis*^19^. Further supporting this hypothesis we here show that the same situation occurs in the rod-shaped Gram-negative *L. pneumophila.* We found that *mreB* was induced because it forms a bicistronic mRNA with *comEA*, a gene coding an essential component of the DNA uptake system. This is most likely due to inefficient transcription termination of the *comEA* gene and may appear as a side effect of *comEA* induction. Yet the co-expression of *comEA* and *mreB* as a bicistronic mRNA suggested a functional significance. For instance, during DNA uptake ComEA relocalises upon DNA binding and this process might have been dependent on MreB dynamics^17^. We first tested whether the increased expression of *mreB* was important for the functioning of the DNA uptake system. By constructing an inducible allele of *mreB* we first found that *mreB* is essential in *L. pneumophila*, confirming it a possible target for antibiotic development^54^. Most importantly for this study we could disconnect *mreB* expression from that of *comEA*. Bacteria expressing limited amount of *mreB*, even below that of non-competent bacteria, remained as transformable as bacteria displaying competence-associated high levels of *mreB*. We could not determine whether MreB showed different localisation pattern in competent cells compared to their non-competent counterparts but large differences in expression levels of *mreB* seemed to have no detectable consequences on growth and morphology of the cells. Although *mreB* expression is required for viability, *mreB* levels below those found in the Paris wild-type strain were sufficient to maintain cell shape and division suggesting that normal MreB levels are not limiting during growth. Growing the *mreB*-inducible strain without inducer we could observe the fate of cells in which MreB levels fall below the level required to maintain the rod-shaped morphology of the bacteria. MreB-depleted bacteria rapidly change morphology and loose viability. Yet before viability is too affected these morphologically-altered cells retained the ability to take up and integrate exogenous DNA. Their transformability is relatively lower (6-fold) but the transformation system seems to remain mostly functional. Mutants of genes involved in the DNA uptake system typically completely lose transformability. For instance, deletion mutants of the *comEA* and *comEC* genes in *L. pneumophila* JR32 strain showed a 1000-fold decreased transformability and no detectable transformability, respectively^39^. If MreB had played an important role in transformation we would have expected a much greater effect but an indirect and secondary role might not be excluded.

The bacterial DNA uptake system is a composite system involving a surface-exposed type IV pilus and a membrane-associated complex that imports the DNA as a single-strand molecule^3^,^32^. In *Pseudomonas aeruginosa* inactivation of MreB leads to mislocalisation of the type IV pilus retraction ATPase PilT leading to a reduction in motility^33^. We cannot exclude the possibility that MreB is required for the proper localisation of the DNA uptake system that most likely involves a type IV pilus in *L. pneumophila*^37^,^55^. This mislocalisation might be responsible for the observed decreased in transformability. Alternatively, MreB has been reported to form membrane regions with increased fluidity which affect the movement of membrane-associated proteins^56^. This could impact the activity of the transmembrane DNA channel for the incoming DNA formed by ComEC. However it is still unclear if MreB-dependent movements of the membrane-associated proteins impact the activity of the membrane-associated processes. The A22 drug is well documented to disrupt the MreB cytoskeleton dynamics at concentrations varying from 10 to 50 μg/mL in multiple species^28^,^33^,^49^,^50^. Yet, A22 could not prevent transformation in *L. pneumophila* when used at concentrations up to 100 μg/mL. At 200 μg/mL a significant decrease in transformation was observed but appeared to be non-specific. Thus A22 could inhibit transformation at high concentrations but it did so independently of MreB. One possibility is that A22 has a secondary target that is involved in natural transformation. A possible candidate is the PilM ATPase which is required for type IV pilus biogenesis and harbors a MreB-like fold^57^.

All the data presented here are consistent with the absence of a direct involvement of MreB in the DNA uptake activity. During the preparation of this manuscript similar conclusions were published in the distantly-related Gram-positive organism *B. subtilis*^34^. The data confirmed that in *B. subtilis mreB* is also induced during competence and under control of the competence regulator ComK^19^,^34^. There are some striking similarity between the two systems. First, ComK does not actually activate transcription of the *mreB* promoter, rather it activates transcription of a probable *maf-radC* operon upstream of *mreB*. Second, like *comEA*, *maf* and *radC* are competence-associated genes. The *radC* gene appears dispensable for transformation in *S. pneumoniae*^9^ and in *H. influenzae* a *radC* mutant shows a small defect in DNA uptake that does not translate into a transformation defect^58^. Nonetheless *radC* is induced during competence in several Gram-negative and Gram-positive organisms. So just like in *L. pneumophila*, the induction of *mreB* is indirect and results from the induction of competence-associated genes located upstream. Similarly to our conclusion in *L. pneumophila*, MreB also plays no direct role in transformation in *B. subtilis*. However MreB appeared to directly or indirectly interact with the ComGA ATPase. In addition to being important for transformation ComGA is responsible for a block of cell division that is noticeable when competent cells developing in stationary phase are placed in fresh medium. While the block of cell division requires Maf and its interaction with ComGA^59^ the sequestration of MreB by ComGA was proposed to prevent cell elongation^34^. A ComGA homolog required for transformation has not yet been identified in *L. pneumophila*. Moreover, in contrast to *B. subtilis*, *L. pneumophila* develops competence in mid-exponential phase and then turns off competence before entering the stationary phase (Fig. 1C,D). So a phenomenon of competence-associated strict inhibition of cell division has not been reported in *L. pneumophila* and it is not known whether this model applies to other competent species. A more detailed analysis of competence development may someday reveal a role of MreB in the timing of competence development or on cell division during this competence phase. Yet on the basis of our work and on the same situation in *B. subtilis*, a direct role of MreB in DNA uptake and transformation can be eliminated.

## Materials and Methods

### Chemicals, media and growth conditions

*L. pneumophila* strains were grown in liquid media ACES [N-(2-acetamido)-2-aminoethanesulfonic acid]-buffered yeast extract (AYE) or on solid media ACES-buffered charcoal yeast extract (CYE) plates. Liquid cultures were performed in 13-mL tube containing 3 mL of medium in shaking incubator at 200 rpm. Alternatively, growth was monitored in 100 μL in 96-well plates placed inside a temperature-controlled plate reader. The plate was submitted to intermittent shaking (100 rpm, 60 sec every 7 min) and growth was monitored by measuring absorbance at 600 nm every 15 min. When appropriate kanamycin and gentamicin were used respectively at 15 μg/mL and 10 μg/mL. Sucrose counter-selection was done on CYE plate containing 5% (w/vol) sucrose. The MreB inhibitor A22 was purchased either from Sigma-Aldrich or Calbiochem, solubilised in dimethyl sulfoxide, aliquoted and stored at −80 °C.

### Strains and plasmids construction

The strains in this study are derived from the *L. pneumophila* Paris clinical isolate (Outbreak isolate CIP107629). Plasmid sequences are available upon request. Oligonucleotides used in this study are listed in Supplementary Table S1. All strain constructions were verified by PCR and sequencing.

Markerless mutants of the *lpp0148* and *lpp2773* genes were constructed in two steps. First a “kan-sacB” cassette carrying a kanamycin resistance gene and the counter-selectable *sacB* gene were inserted in the *lpp0148* and *lpp2773* genes. To do so the upstream and downstream regions (2 kb each) of *lpp0148* and *lpp2773* were amplified with primers carrying 30-nucleotide sequences complementary to ends of the *kan-sacB* cassette. The upstream and downstream region were assembled to the *kan-sacB* cassette by PCR overlap extension (PrimeSTAR Max polymerase, Takara) and used to transform the Paris wild-type strain by natural transformation followed by selection on kanamycin^41^. The interrupted *lpp0148* and *lpp2773* genes were then replaced by alleles in which an ochre stop codon (TAA) was introduced between the eleventh and twelfth codons. The mutated alleles were synthesised by overlapping PCR and introduced in the strains by natural transformation^39^ followed by selection on 5% sucrose. Sucrose-resistant and kanamycin-sensitive colonies carried the alleles of *lpp0148*_*TAA*_ and *lpp2773*_*TAA*_ containing the premature stop codon.

The inducible mutant of *mreB* was obtained by replacing its promoter by a synthetic IPTG-inducible promoter at the natural chromosomal locus in the *lpp0148*_*TAA*_ mutant strain. A synthetic cassette was constructed to carry the *lacIq* gene, followed by a gentamicin resistance gene and a synthetic promoter with a –35 box (TTGCTT) and a –10 box (TATAAT) each followed by a LacO operator site (ATTGTGAGCGGATAACAATT). The cassette was integrated between the *comEA (lpp0872*) and *mreB (lpp0873)* gene by natural transformation as described above. Transformants were selected on CYE plates containing gentamicin and IPTG (0.5 mM). The resulting *lpp0148*_*TAA*_ *mreB*^*ind*^ strain requires IPTG for growth in liquid and solid media.

The A22 resistant alleles of *mreB* were transferred to a new genetic background by natural transformation of the PCR-amplified *mreB* alleles in the *lpp0148*_*TAA*_ *mreB*^*ind*^ strain. Colonies that could grow in the absence of IPTG had acquired the A22-resistant *mreB* allele and lost the *lacIq* and gentamicin resistance cassette.

The pGEM-ihfB::Kan plasmid used in natural transformation experiments was constructed by cloning a 5 kb fragment containing *ihfB* interrupted by a kanamycin resistance gene^60^ into the pGEM-T easy vector (Promega).

### RNA extraction

Bacteria were grown in AYE to the desired optical density and cells were fixed by adding 1 mL of culture to a 2-mL tube on ice containing an equal volume of methanol. The mixed suspension was kept on ice until the bacterial cells were collected by centrifugation (1 min, 21,000 g, 4 °C) and then stored at −80 °C. Thawed bacterial pellets were resuspended in 50 μL of RNAsnap buffer (18 mM EDTA, 0.025% SDS, 1% 2-mercaptoethanol, 95% formamide)^61^ and lysed by incubating the suspension at 95 °C for 7 min. One millilitre of commercial tri-reagent solution (acid guanidinium thiocyanate-phenol-chloroform)^62^ was added and RNA extraction was performed following the manufacturer’s instructions. RNA sample purity and concentration were determined by spectrophotometric analysis on a NanoDrop 2000 UV-Vis Spectrophotometer (Thermo).

### Northern-blot

Northern-blot analysis were performed as previously described^39^. Briefly, three micrograms of total RNA in denaturing buffer were loaded per lane and run on denaturing formaldehyde 1.5% agarose gel in MOPS buffer. RNA were transferred to nylon membrane (IMMOBILON-NY+, Millipore corporation) by capillary transfer in 10X SSC buffer (Saline Sodium Citrate 1X: 150 mM NaCl + 15 mM sodium citrate) and then cross-linked to the membrane by UV irradiation. Membrane were hybridised at 42 °C with 5′-biotinylated oligonucleotide probes (5 nM) for either *comEA* (comEA2-NB) or *mreB* (mreB-NB) in ULTRAhyb Ultrasensitive Hybridisation Buffer (Ambion, Austin, TX). Following overnight hybridisation the membranes were washed twice in 2X SSC buffer containing 0.1% SDS at 65 °C according to the ULTRAhyb manufacturer instructions. Probed membranes were revealed using horseradish peroxidase-conjugated streptavidin and enhanced luminol substrate (Chemiluminescent Nucleic Acid Detection Module, Pierce, Rockford, IL). Luminescence signals were acquired using an imaging workstation equipped with a charge-coupled device camera (Thermo).

### Determination of natural transformability

The transformation ability of *L. pneumophila* Paris wild-type strain at different time points during growth was determined as follows. The strain was streaked on CYE solid medium and plates were incubated overnight at 37 °C. Bacteria were then resuspended in AYE and this suspension was used to inoculate 50 mL AYE at a starting OD_600_ of 0.05 in 500-mL Erlenmeyer flasks. Cultures were incubated under constant shaking at 30 °C and, when an OD_600_ of interest was reached, the volume containing 1.10^9^ cells was collected, centrifuged for 3 minutes at 5000 g and pellets were resuspended in 200 μL AYE. This cell suspension was mixed with 3 μg of pGEM-ihfB::Kan and incubated 30 min at 30 °C without shaking (this plasmid is non-replicative in *L. pneumophila* but contains a kanamycin-resistance cassette inserted in the *ihfB* gene of *L. pneumophila*, this locus can recombine with the chromosome and this produces kanamycin-resistant transformants). 20 U of DNase I (Sigma) was added and the volume was adjusted to 1 mL with AYE. The cells were incubated 2 h at 37 °C shaking and ten-fold serial dilutions were then plated on non-selective medium (CYE) and selective medium (CYE + Kanamycin 15 μg/mL). Plates were incubated for 72 h at 37 °C and colony-forming units (CFU) counting was performed. Transformation frequency is the ratio of the number of CFUs counted on selective medium divided by the number of CFUs counted on non-selective medium.

The transformation ability of the *lpp0148*_*TAA*_ *mreB*^*ind*^ strain compared to the *lpp0148*_*TAA*_ strain was determined as follows. The *lpp0148*_*TAA*_ *mreB*^*ind*^ strain was streaked on CYE solid medium containing IPTG at 20 μM. The *lpp0148*_*TAA*_ strain was streaked on CYE solid medium without IPTG. Plates were incubated overnight at 37 °C, and bacteria were resuspended at an OD_600_ = 0.2 in 2 mL AYE, containing 20 or 500 μM IPTG. Tubes were incubated under constant shaking at 37 °C. When an OD_600_ of approx. 1 was reached, 1 mL of culture was centrifuged for 3 min at 5000 g in a table-top microcentrifuge, and pellets were resuspended in 200 μL of distilled water containing 2 μg of pGEM-ihfB::Kan. After 20 minutes of incubation at 37 °C without shaking, tubes were centrifuged 3 minutes at 5000 g and pellets were washed twice in AYE. Pellets were finally resuspended in 200 μL AYE. Ten-fold serial dilutions were then plated on non-selective medium (CYE, with 20 μM IPTG when appropriate) and selective medium (CYE + Kanamycin 15 μg/mL, with 20 μM IPTG when appropriate). Plates were incubated for 72 h at 37 °C and transformation frequencies were determined as previously described. The effect of A22 inhibitor on transformation was determined by adding A22 at the indicated concentration along with the transforming DNA.

The transformation ability of the *lpp0148*_*TAA*_ *mreB*^*ind*^ strain in the absence of IPTG was determined similarly except that centrifugation steps were avoided to preserve bacterial morphology. Transformation experiments were done using 1 mL of culture and 5 μg pGEM-ihfB::Kan. The *lpp0148*_*TAA*_ *mreB*^*ind*^ strain was streaked on CYE medium containing 20 μM IPTG and incubated overnight at 37 °C. The strain was then resuspended in AYE media at an OD_600_ = 0.2 and incubated under constant shaking at 37 °C. After 0, 2, 4, 6 and 8 hours, 1050 μl of culture was taken and put into contact with 5 μg of pGEM-ihfB::Kan for 20 minutes at 37 °C. After 20 min of incubation at 37 °C, 50 μL of the suspension were used to perform ten-fold serial-dilutions which were then plated on selective and non-selective medium (CYE + kanamycin 15 μg/mL + 20 μM IPTG, and CYE + 20 μM IPTG, respectively) and the remaining culture (~1 mL) was used to flood a plate of selective medium. Plates were incubated 72 h at 37 °C and transformation frequencies were determined as previously described. At each tested time, OD_600_ of the culture was measured, and 1 mL of culture was used to perform microscopic observation of the bacteria as described further below.

### Isolation of A22-resistant mutants

A22-resistant mutants were obtained by plating a stationary culture of the *lpp0148*_*TAA*_ strain on CYE medium containing A22 at 25, 50 or 100 μg/mL. After 72 h of incubation at 37 °C, single colonies were picked and streaked on CYE medium. After 48 h of incubation at 37 °C, mutants were collected and stored in AYE medium with 15% glycerol at −80 °C. We analysed 4, 8 and 2 mutants obtained on plate containing A22 at 25, 50 and 100 μg/mL, respectively. Sequencing confirmed that the selected mutants each harboured mutations in the *mreB* gene (Fig. S2). Four mutations conferring high resistance levels to A22 were transferred to a clean *lpp0148*_*TAA*_ genetic background using the *lpp0148*_*TAA*_ *mreB*^*ind*^ strain. Mutated *mreB* genes of the A22 spontaneous-resistant mutants were amplified using mreBseqF and mreBseqR primers. The PCR product was then added to a 200 μL AYE culture of the *lpp0148*_*TAA*_ *mreB*^*ind*^ strain that requires IPTG for *mreB* expression and growth. After 20 minutes of incubation, bacteria were plated on CYE medium without IPTG. After 72 h of incubation at 37 °C, we obtained colonies that grew in the absence of IPTG implying a loss of the inducible promoter and potentially a transformation by the mutated *mreB* gene. Sequencing confirmed the acquisition of the mutated *mreB* allele in an otherwise wild-type locus. The four *mreB* mutants reconstructed this way were tested for A22 resistance and used in natural transformation experiments.

### Determination of A22-resistance by disc diffusion assay

Strains of interest were resuspended at an OD_600_ = 0.1 in 3 mL AYE liquid medium and incubated for two days at 37 °C under constant shaking. The culture was used to flood a CYE plate for a few minutes. Liquid medium was then removed and plates were dried at room temperature for 30 min. A paper disc impregnated with 10 μL of an A22 solution (10 mg/mL) was then placed at the centre of each plates. Plates were incubated 3 days at 37 °C before reading.

### Microscopy

Bacteria were collected from 1 mL of culture by centrifugation 3 min at 5000 g and pellets were resuspended in 300 μL PBS Formaldehyde 3.7% and incubated at room temperature for 30 min. Acid-washed (ethanol/HCl 1 M) glass coverslips were coated with poly-L-lysine by immersion in a poly-L-lysine 0.01% solution in distilled water (Sigma-Aldrich) for 5 min. Fixed bacteria in PBS Formaldehyde 3.7% were pipetted on the air-dried coverslips and let to settle and stick to the coverslips for about 30 min. Coverslips were then washed twice with PBS, and DNA was labelled using Hoechst 33288 (12 μg/mL in PBS) for 1 h at room temperature. Coverslips were washed twice in PBS and mounted using 8 μL of mounting solution (DAPCO). After an overnight incubation at 4 °C, slides were observed and imaged with an epifluorescence microscope (Zeiss Axioplan 2). Morphological changes observed in the *lpp0148*_*TAA*_ *mreB*^*ind*^ strain when grown in the absence of IPTG were quantified by measuring the width of bacterial cells at mid-cell. The measurement of bacterial cells width was performed on pictures from the microscopy experiments, using the KLONK Image Measurement software. At least one thousand bacterial cells were measured for each condition. A Shapiro-Wilk test confirmed the Normal distribution of the measured parameter for each condition (p < 0.01). Significance testing was performed using a Welsh t-tests and two samples with a P value < 0.01 were considered significantly different.

## Additional Information

**How to cite this article**: Juan, P.-A. *et al.* Natural transformation occurs independently of the essential actin-like MreB cytoskeleton in *Legionella pneumophila*. *Sci. Rep.* **5**, 16033; doi: 10.1038/srep16033 (2015).

## Supplementary Material

Supplementary Information
