# Supplementary material for: Natural transformation occurs independently of the essential actin-like MreB cytoskeleton in Legionella pneumophila
Source: Sci Rep. 2015 Nov 3;5:16033. doi: 10.1038/srep16033 (PMC4630621; doi:10.1038/srep16033)
Supplement: Supplementary Information [file srep16033-s1.pdf]

Supplementary Material

Natural transformation occurs independently of the essential actin-like MreB cytoskeleton in *Legionella pneumophila*

Pierre-Alexandre Juan[1,2], Laetitia Attaiech[1,2], and Xavier Charpentier[1,2]*

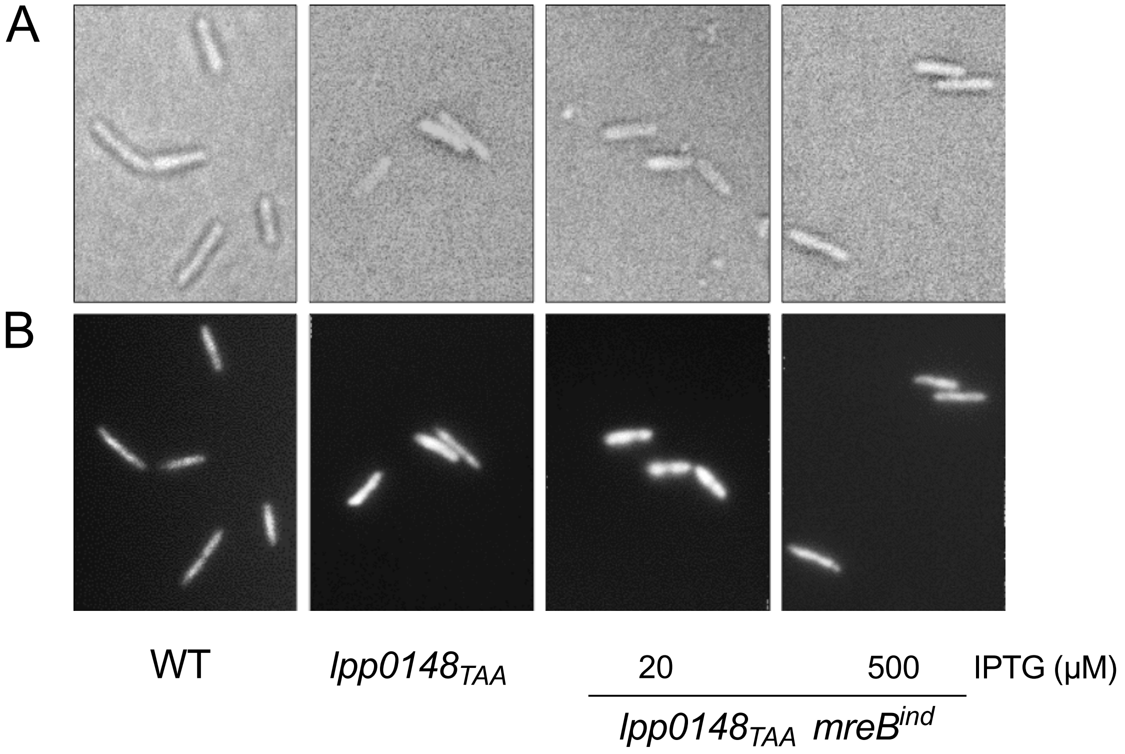

Supplementary Figure S1. Microscopy of *L. pneumophila* cells expressing various levels of *mreB* (see Figure 3). Bacteria were grown to exponential phase (OD 600nm = 1), fixed in 3% formaldehyde and DNA was stained with Hoescht 33288. A. Observation under bright light. B. Fluorescence imaging of DNA staining.

A

L. pneumophila Paris		Isolated on
CDS mutation	AA mutation	[A22] (μg.ml^{-1})
G725A	**G242D**	25
T71C	**I24T**	50
A220C T221A	**M74Q**	50
T244C	**F82L**	50
C424G	**P142A**	50
G886A	**A296T**	50
T245C	**F82S**	100
C338T	**P113L**	100

B

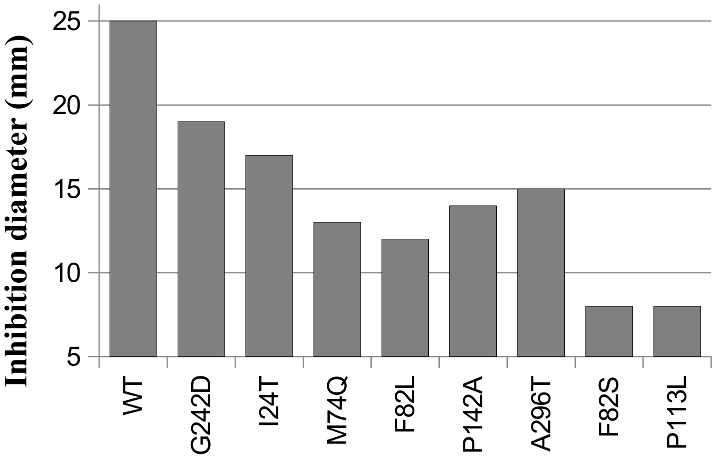

C

```
Lp    1 MFRKLRGVFSSDLSI DLGTANTLIYV RDKGIVLNEPSVVALRNE--SGQKRV  50
Ec    1 MLKKFRGMFSNDLSI DLGTANTLIYV KGQGIVLNEPSVVAIRQDRAGSPKSV  52
Vc    1 MFKKLRGMFSNDLSI DLGTANTLIYV KGQGIVLDEPSVVAIRQDKGRGGKTV  52
Cc    1 MFSSLFGVISNDIAI DLGTANTLIYQ KGKGIVLNEPSVVALRNV--GGRKVV  50
Pa    1 MFKKLRGMFSSDLSI DLGTANTLIYV RERGIVLNEPSVVAIRSH--GSQKSV  50

Lp   51 AAVGLEAKRMLGRTPGNINAI RPMKDGVIADF FVTEKMLQHFIHKVHENK 100
Ec   53 AAVGHDAKQMLGRTPGNIAAI RPMKDGVIADF FVTEKMLQHFIKQVHSNS 102
Vc   53 AAVGHAAKQMLGRTPGNISAI RPMKDGVIADF YVTEKMLQHFIRQVHDNS 102
Cc   51 HAVGIEAKQMLGRTPGHMEAI RPMRDGVIADF EVAEEMIKYFIRKVHNRK 100
Pa   51 VAVGTEAKRMLGRTPGNIAAI RPMKDGVIADF SVCEKMLQYFINKVHENS 100

Lp  101 FLRPSPRVLV CVP CGSTQVERRA I RESAMGAGAREVFLIE EP MAAALGSG 150
Ec  103 FMRPSPRVLV CVP VGATQVERRA I RESAQGAGAREVFLIE EP MAAAIGAG 152
Vc  103 VLKPSPRVLV CVP CGSTQVERRA I RESALGAGAREVYLID EP MAAAIGAG 152
Cc  101 GF-VNPKVIV CVP SGATAVERRA I NDSCLNAGARRVGLID EP MAAAIGAG 149
Pa  101 FLQPSPRVLI CVP CKSTQVERRA I RESALGAGAREVFLIE EP MAAAIGAG 150

Lp  151 MPVEEASGSMVV DIGGGTTEVA IISLSGIVY HQSVRIGGDKFD DAIVSYV 200
Ec  153 LPVSEATGSMVV DIGGGTTEVA VISLNGVVY SSSVRIGGDRFD EAIINYV 202
Vc  153 LRVSEPTGSMVI DIGGGTTEVA VISLNGVVY SSSVRIGGDRFD EAIINYV 202
Cc  150 LPIHEPTGSMVV DIGGGTTEVA VLSLSGIVY SRSVRVGGDKMD EAIISYM 199
Pa  151 LPVEEARGSMVV DIGGGTTEIA LISLNGVVY AESVRVGGDRFD EAIVTYV 200

Lp  201 RRNYGTLIGETTA ERIKH EIGSA-FPS-RDLFEIEVRGRNLAE G VPRSFTLT 250
Ec  203 RRNYGSLIGEATA ERIKH EIGSA-YPG-DEVREIEVRGRNLAE G VPRGFTLN 252
Vc  203 RRNYGSLIGEATA EKIKH EIGSA-YPG-DDVQEIEVRGRNLAE G VPRSFTLN 252
Cc  200 RRHHNLLIGETTA ERIKK EIGTARAPADGEGLSIDVKGRDLMQ G VPREVRIS 251
Pa  201 RRNYGSLIGESTA ERIKQ EIGTA-FPG-GDVREVDVRGRNLAE G VPRSFTLN 250

Lp  251 SAEILEALQEPLSGIVGAVRA A LELAPPELAADIAERGMVLTGG GA LLKN 300
Ec  253 SNEILEALQEPLTGIVSAVMV A LEQCPPELASDISERGMVLTGG GA LLRN 302
Vc  253 SNEILEALQEPLTGIVSAVMV A LEQCPPELASDISENGMVLTGG GA LLKD 302
Cc  252 EKQAADALAEPVGQIVEAVKV A LEATPPELASDIADKGIMLTGG GA LLRG 301
Pa  251 SNEVLEALQESLATIVQAVKS A LEQSPPELASDIAERGLVLTGG GA LLRD 300

Lp  301 IDTLLMEETGLPVLIAED PLTCV ARGGGKALETMDLRGGDFLSTE 345
Ec  303 LDRLLMEETGIPVVVAED PLTCV ARGGGKALEMIDMHGGDLFSEE 347
Vc  303 LDRLLMEETGIPVVIADD PLTCV ARGGGKALEMIDMHGGDLFSEE 347
Cc  302 LAEIRDHTGLPVTVADDP LSCVA LGCGKVLEHPKWMKGVLESTLA 346
Pa  301 LDKLLAQETGLPVIVAEE PLTCV ARGGGRALEMMDRHSMDLLSTE 345
```

Supplementary Figure S2. A. Table of *mreB* spontaneous mutations obtained while selecting for A22-resistant mutants. B. A22 susceptibility of the A22-resistant MreB mutants determined by disk diffusion assay. C. Alignment of the amino acid sequence of MreB protein of *L. pneumophila* Paris (Lp) with sequences of its homologs in *E. coli* (Ec), *V. cholerae* (Vc), *C. crescentus* (Cc) and *P. aeruginosa* (Pa). The amino acids altered in A22-resistant MreB mutants are presented on a black background (for *L. pneumophila*) or gray background (for the other species). Alterations positions are boxed for easier identification. For each species, the ATP binding pocket is shown by the amino acids colored in red (only for *L. pneumophila*).

Natural transformation occurs independently of the essential actin-like MreB cytoskeleton in *Legionella pneumophila*

Supplementary table S1 – List of primers used in this study

Primers for the construction of the lpp0148 and lpp2773 mutant strains

Name	Description/Use	Sequence (5 '- 3')
lpp0148_P1	Forward primer to amplify a 2kb fragment upstream of lpp0148	GCCGTTTTAAATCGGCCAGAAAG
lpp0148_P2	Reverse primer to amplify a 2kb fragment upstream of lpp0148. Underlined sequence is complementary to the 5' end of the kan-sacB cassette	GGCCCAATTCGCCCTATAGTGAGTCGCAGGCAAGCCAGATAATCAAGAC
lpp0148_P3	Forward primer to amplify a 2kb fragment downstream of lpp0148. Underlined sequence is complementary to the3' end of the kan-sacB cassette	GGGTTTGCTCGGGTCGGTGGCATATGAAAGCTCGTGAAGTCCGTATCG
lpp0148_P4	Reverse primer to amplify a 2kb fragment downstream of lpp0148.	CTTCCCCCTAAAAATCAGGATGTC
lpp0148TAA_P2	Reverse primer used with lpp0148_P1 to amply the 5' end of lpp0148	GCTTTATTGATGAC**TTA**AGCGGTTCGTGGG
lpp0148TAA_P3	Foward primer used with lpp0148_P4 to amply the 3' end of lpp0148	CCCACGAACCGCT**TAA**GTCATCAATAAAGC
lpp2773_P1	Forward primer to amplify a 2kb fragment upstream of lpp2773	GGCGATTTCGCATGTAATGCTC
lpp2773_P2	Reverse primer to amplify a 2kb fragment upstream of lpp2773. Underlined sequence is complementary to the 5' end of the kan-sacB cassette	GGCCCAATTCGCCCTATAGTGAGTCGGCCATCCTGGCGAAACATTTAG
lpp2773_P3	Forward primer to amplify a 2kb fragment downstream of lpp2773. Underlined sequence is complementary to the3' end of the kan-sacB cassette	GGGTTTGCTCGGGTCGGTGGCATATGTTTTGTTTGGTGAAACCCCTAATG
lpp2773_P4	Reverse primer to amplify a 2kb fragment downstream of lpp2773.	ACGCTTGTTCGATTGCTGGGTG
lpp2773TAA_P2	Reverse primer used with lpp2773_P1 to amply the 5' end of lpp2773	CGCAAGCGGTA**TTA**AGCAAACTGTTTTG
lpp2773TAA_P3	Foward primer used with lpp2773_P4 to amply the 3' end of lpp2773	CAAAACAGTTTGCT**TAA**TACCGCTTGCG
lpp0148seqF	Sequencing primer for verification of the mutant strain	CTATCATACATTCAAGGTCAACAGACC
lpp0148seqR	Sequencing primer for verification of the mutant strain	TTTGGCGCAGCCCATAATGGAG
lpp2773seqF	Sequencing primer for verification of the mutant strain	CCTTCAAGTAACATCAGCGTCCG
lpp2773seqR	Sequencing primer for verification of the mutant strain	GAGTGGAGCACTATCTCTTTTGAGC

Primers for the construction of the mreB mutant strain

Name	Description/Use	Sequence (5 '- 3')
mreBseqF	Forward primer for sequencing of *mreB* (lpp0873)	CCGGTTAAAAGCAGTTGTCTGG
mreBseqR	Reverse primer for sequencing of *mreB* (lpp0873)	CCCAGAGAACTGTGTCCGCCC
mreBseq_Fw	Forward primer to sequence *mreB* (lpp0873)	GTATAAAATTCTTATGCGGCAACTGTC
mreBseq_IntFw	Forward primer to sequence *mreB* (lpp0873)	GTCGCAATTATTTCACTAAGTGGAATAG
lpp0873_P1	Forward primer to amplify a 2kb fragment upstream of *mreB* (lpp0873)	CCAGGGATTGGGAATAAGCGTTG
0873-ptac-P2	Reverse primer to amplify a 2kb fragment upstream of *mreB* (lpp0873). The underlined sequence is complementary to 5' end of the gentamicin resistance gene	TTCCACGGTGTGCGTCCATGGGCAATTGCCGCATAAGAATTTTATACACC
gnt-F	Forward primer to amplify a 2.3kb cassette containing the gentamicin resistance gene and the lacIq gene	TTGCCCATGGACGCACACCGTG
lacIq-R1	Reverse primer to amplify a cassette containing the gentamicin resistance gene and lacIq gene. The underlined sequence is complementary to the IPTG-inducible promoter	ATTATAATTGTTATCCGCTCACAATAAGCAAAGAACCGTTATGATGTCGGCGC
0873-ptac-P3	Forward primer to amplify a 2kb fragment starting at the *mreB* (lpp0873). The primer carries the IPTG-inducible promoter. The underlined sequences are lacO sites and -35 and -10 box are in bold.	**TTGCTT**ATTGTGAGCGGATAACAAT**TATAAT**TGTGAGCGGATAACAATTCTGTCGATTTTTATCCAAATATAGG
lpp0873_P4	Reverse primer to amplify a 2kb fragment downstream of *mreB* (lpp0873)	GGCGAATTACCCGAACAAACTCTC

Probes for Northen-blot

Name	Description	Sequence (5' - 3')
comEA2-NB	5' and 3' biotinylated probe for the detection of the *comEA* mRNA	CTACAAAACGCTGCCCTATACCCTTGACTTCCGCAAGCTCTTCCAGAG
mreB-NB	6' and 3' biotinylated probe for the detection of the *mreB* mRNA	CCTACAGCGGCGACACGCTTCTGGCCTGATTCATTACGCAATGCC
